# Exploring *THAP11* Repeat Expansion beyond Chinese‐Ancestry Cohorts: An Examination of 1000 Genomes and UK Biobank Data

**DOI:** 10.1002/mds.29636

**Published:** 2023-12-19

**Authors:** Liam G. Fearnley, Haloom Rafehi, Mark F. Bennett, Melanie Bahlo

**Affiliations:** ^1^ Population Health and Immunity Division The Walter and Eliza Hall Institute of Medical Research Parkville Victoria Australia; ^2^ Department of Medical Biology The University of Melbourne Parkville Victoria Australia; ^3^ Bruce Lefroy Centre for Genetic Health Research Murdoch Children's Research Institute Parkville Victoria Australia; ^4^ Epilepsy Research Centre, Department of Medicine University of Melbourne, Austin Health Heidelberg Victoria Australia

**Keywords:** ataxia, population studies, biobanks, repeat expansions, bioinformatic screening

Tan et al report novel CAG repeat expansion in *THAP11* associated with spinocerebellar ataxia (SCA) in two Chinese families. They observed 45–100 repeats, three CAA interruptions, and a long uninterrupted 3’ tail in sequencing of ataxic individuals from a single family.^1^

We investigated presence and size of *THAP11* expansion in short‐read next‐generation sequencing of individuals with other ancestries in 1000 Genomes and the UK Biobank.^2^ Repeat genotypes of up to 50 triplets can be accurately typed with short read sequencing and bioinformatic tools.^3^

## Methods

We genotyped CAG expansion in *THAP11* (GRCh38 position chr16:67842863‐67842950) with ExpansionHunter 5.0.0 and REViewer^4^ in 138 individuals with whole‐genome sequencing in the UK Biobank^2^ and a history of hereditary ataxia or ataxia of unknown cause (ICD10 G110‐G119; R270). We obtained *THAP11* ExpansionHunter genotypes from 2504 unrelated individuals in 1000 Genomes.^5^

## Results

**FIG. 1:**
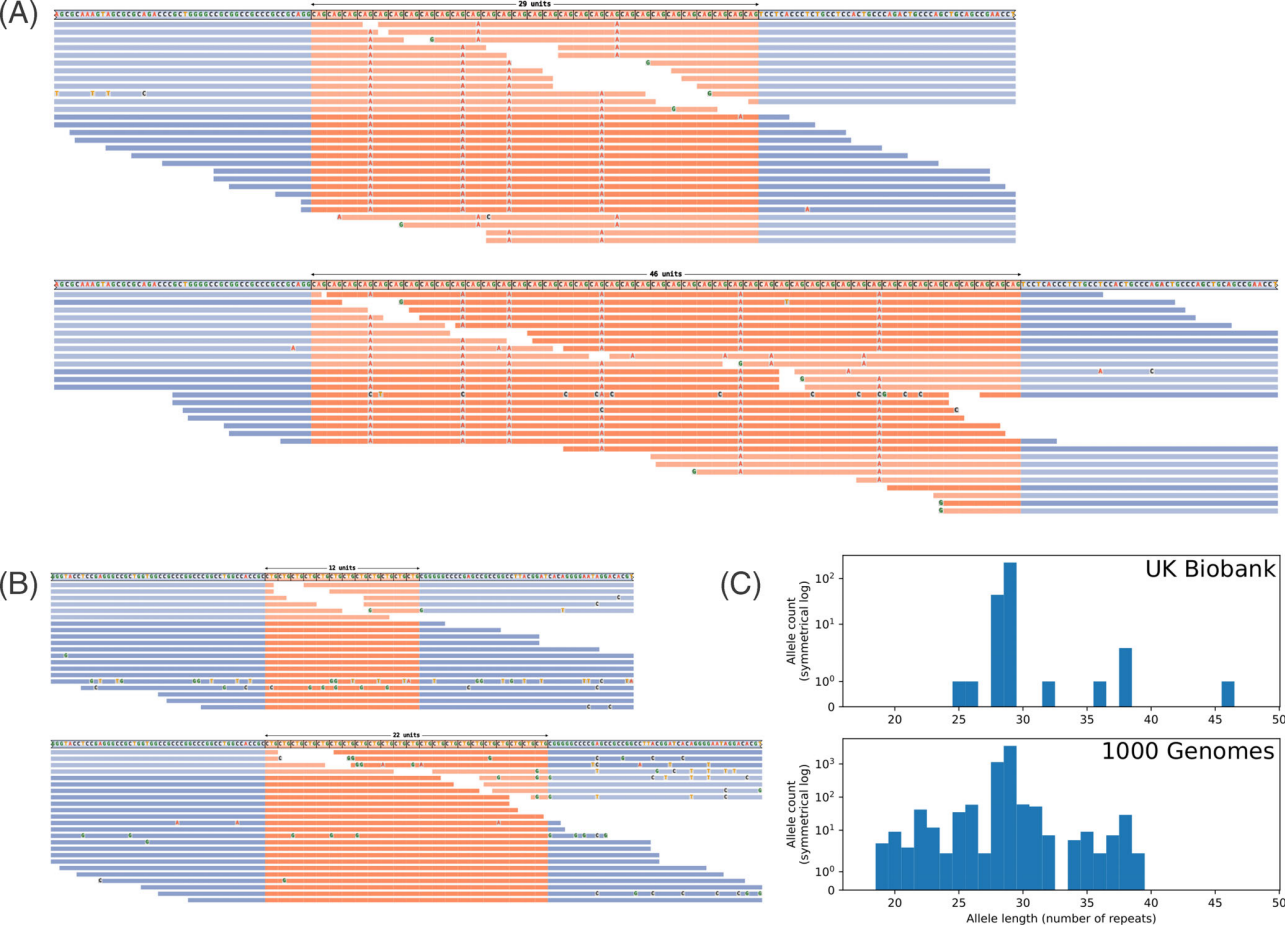
(**A**) REViewer plot of *THAP11* CAG expansion in a European ancestry participant in the UK Biobank. CAA interruptions are present in the expanded allele. (**B**) REViewer plot of expanded *CACNA1A* allele in the same individual. (C) Distribution of *THAP11* allele sizes in 139 individuals with ataxia in the UK Biobank and 2504 unrelated individuals in the 1000 Genomes cohort.

One European‐ancestry ataxic individual in the UK Biobank has a 46/29 CAG *THAP11* genotype, exceeding the proposed pathogenic threshold,^1^ and an uninterrupted 22‐repeat CAG repeat expansion in *CACNA1A* that likely causes spinocerebellar ataxia type 6 (SCA6).^6^ REViewer visualization of ExpansionHunter genotyping shows six CAA interruptions to the *THAP11* expansion (Fig. 1A) and no interruptions in the SCA6 expansion (Fig. 1B).

The individual had primary care diagnoses of hereditary ataxia at 40, Parkinson's disease at 53, and received repeated prescriptions for trihexyphenidyl, a drug used in management of movement disorders. More‐precise age of onset is not available as medical records in the UK Biobank are partial and participants are not re‐contactable.

*THAP11* genotypes in the 1000 Genomes cohort were between 19 and 39 repeats. In the UK Biobank ataxia cohort, the range was 25 to 46 repeats. In both cohorts, the median was 29 repeats (IQR, 28–29) (Fig. 1C).

## Discussion

We report the first finding of *THAP11* CAG expansion in an ataxic individual of European ancestry. Interpretation of this expansion is complicated by detection of an uninterrupted pathogenic full‐penetrance length SCA6 expansion and diagnosis of both ataxia and Parkinson's disease. This individual has six CAA interruptions, with nine uninterrupted 3′ repeats. We also provide length distributions of the *THAP11* allele.

Tan et al suggest toxicity of CAA‐interrupted repeats based on CAG‐pure sequences of ataxic individuals. They report one family where ataxic individuals with *THAP11* expansion have three interruptions and 32 to 87 uninterrupted repeats in the 3′ end of the expansion, and unaffected family members have five to six interruptions and shorter tails. They also report *THAP11* expansion in an unrelated individual (patient II‐1), with six interruptions and 10 uninterrupted 3′ repeats (Tan et al's supporting information Figure S3),^1^ similar to our European‐ancestry individual.

Studies suggest CAA interruptions to CAG expansions stabilize intergenerational variability in repeat length,^7^ which may contribute to expansion instability in the family with fewer interruptions. Our findings show further work is necessary to elucidate the role of rare *THAP11* expansion and its composition in ataxia and demonstrates that *THAP11* expansion is detectable with bioinformatic approaches. It also further highlights the emerging complexity of expansion composition in tandem repeat‐mediated disease.

## Financial Disclosures

This work was supported by the National Health and Medical Research Council (NHMRC) grants (GNT2001513, MRFF2007707 and MRFF2007677) to M.B., M.F.B. and H.R. H.R. was supported by a NHMRC Emerging Leadership 1 grant (1194364) and M.B. was supported by a NHMRC Leadership 1 grant (1195236). Additional funding was provided by the Independent Research Institute Infrastructure Support Scheme and the Victorian State Government Operational Infrastructure Program.

## Author Roles

(1) Research project: A. Conception, B. Organization, C. Execution; (2) Statistical Analysis: A. Design, B. Execution, C. Review and Critique; (3) Manuscript: A. Writing of the First Draft, B. Review and Critique.

L.G.F.: 1A, 1B, 1C, 2A, 2B, 3A, 3B; H.R. 1A, 2B, 2C, 3B; M.F.B. 1A, 2C, 3B; M.B. 1A, 1B, 2A, 2C, 3B.

## Data Availability

The data that support the findings of this study are available from the UK Biobank. Restrictions apply to the availability of UK Biobank data. Data from the Illumina Repeat Catalog are openly available at https://github.com/Illumina/RepeatCatalogs.
